# Supplementary material for: Regulatory mechanisms underlying coordination of amino acid and glucose catabolism in Escherichia coli
Source: Nat Commun. 2019 Jul 26;10:3354. doi: 10.1038/s41467-019-11331-5 (PMC6659692; doi:10.1038/s41467-019-11331-5)
Supplement: Supplementary file 1 — Supplementary Information [file 41467_2019_11331_MOESM1_ESM.pdf]

Supplementary Information

of

Regulatory mechanisms underlying coordination of amino acid and glucose catabolism in *Escherichia coli*

Authors: Mattia Zampieri[1*]†, Manuel Hörl[1]†, Florian Hotz[1], Nicola F. Müller[1] and Uwe Sauer[1*].
(† *equal contribution*)

Affiliations:

[1]Institute of Molecular Systems Biology, ETH Zürich, Switzerland.

*To whom correspondence should be addressed: zampieri@imsb.biol.ethz.ch

Supplementary Discussion

Metabolome changes upon perturbation with different sets of amino acids

Notably, different rates of glucose uptake and acetate secretion, were not the only difference observed among the different amino acid perturbations (Supplementary Figure **15**):

- Both CAA and SAA addition induced an accumulation of several TCA cycle intermediates, mainly within the non-oxidative branch (succinate, fumarate, malate and oxaloacetate). On the contrary, intermediates in the oxidative branch of the TCA cycle and upper glycolysis, such as citrate, aconitate, α-ketoglutarate or fructose 1-6 bisphosphate showed only minor changes over time. We also observed prolonged accumulation of intermediates within nucleotide metabolism such as UMP, GMP, guanine and deoxyadenosine. Amongst all metabolites, pyruvate exhibited unique dynamic changes, undergoing fast accumulation upon CAA addition (i.e. 25-fold higher) within the first 30 minutes, before coming back at 90 minutes to basal levels.
- Only when tryptophan was excluded from the perturbing amino acid mix, UMP and indole were not accumulating. Tryptophan and UMP biosynthesis share 5-phospho-α-D-ribose 1-diphosphate as a common precursor. When tryptophan is supplemented in the medium, cells seem to overproduce UMP. Similarly, indole is the direct precursor of tryptophan and the degrading enzyme TrpB is inhibited by tryptophan [1].
- The presence or absence of glutamate in the amino acids mix is clearly visible from the presence/absence of accumulating 4-aminobutanoate, part of the glutamate dependent acid resistance mechanism [2].

- When cells are perturbed with amino acid mix lacking aspartate and asparagine, we observed significant differences within TCA cycle intermediate profiles, which were again in agreement with model predictions. Contrary to all other conditions, TCA cycle intermediates are not accumulating, except for pyruvate, which showed similar fast accumulation, but final levels after 90 minutes from the addition of amino acids lower than the initial steady state levels. This observation is also consistent with the previous observation of an aspartate dependent activation of malic enzymes (Figure 2 of the main text).
- We only observed a reduction in both glucose uptake repression and acetate overflow when all amino acids degradable into pyruvate were removed from the synthetic amino acid mix (Supplementary Data 1, Supplementary Figure 15). Deprivation of either Serine/Threonine/Glycine or Serine/Threonine/Glycine/Tryptophan/Alanine (i.e. SAA-STG and SAA-STGTCA) also caused a clearly reduced accumulation of pyruvate, in agreement with model predictions of high catabolism of serine, glycine and threonine (Supplementary Figure 10).

Overall, we revealed perturbation specific metabolic responses, where central carbon intermediates were mainly affected by catabolism of serine/glycine/threonine into pyruvate, and of aspartate/asparagine into oxaloacetate, consistent with model flux predictions of high catabolic fluxes for these amino acids. In fact, pyruvate and oxaloacetate are the compounds undergoing the most significant changes when cells are perturbed with different classes of degradable amino acids. In particular, direct catabolism of serine/glycine/threonine seems to be responsible for the initial accumulation of pyruvate. On the other hand, aspartate/asparagine metabolism triggers a sequential chain of accumulation among the intermediates of the non-oxidative branch of TCA cycle, in parallel to a more prolonged accumulation of pyruvate.

Supplementary Methods

Identification of metabolites within casamino acids

To identify compounds besides amino acids, which did not originate from cell culture, but were already present in the casamino acids powder (CAA), we profiled glucose minimal medium, supplemented with 6 different concentrations of either CAA (i.e. 2-1-0.5-0.25-0.125-0.0625 g/L.) or a synthetic amino acid mixture (SAA) by time of flight mass spectrometry [3]. 167 out of the total 427 metabolites, detected in culture supernatants, were found to have a linear relationship between CAA concentration and measured intensities (Supplementary Figure 2). Besides amino acids, we detected the presence of several nucleotide precursors, such as dethiobiotin, uridine, xanthosine or GMP in CAA medium, but not in SAA medium (Supplementary Data 1). To estimate a plausible range of concentrations for the 167 compounds, we selected uridine, citrate and GMP as representative cases. These compounds showed radically different fold-changes with respect to the reference base-line samples (from 300 to 4-fold higher respectively, Supplementary Figure 2B), hence providing us with

qualitative upper and lower bounds for the other detected compounds. For these 3 compounds we estimated absolute concentrations by using pure external standards. We estimated concentrations ranging in the µM range of: 121 ± 0.0077 for uridine, 30±0.1 for citrate and 71 ± 0.0402 for GMP.

To test, whether the detected compounds were present in biologically significant concentrations, we monitored growth of single gene knockouts auxotrophic for: uridine (ΔpyrE), biotin (ΔbioA, ΔbioB, ΔbioD) or xanthine/xanthosine (ΔpurC, ΔpurE, ΔpurH, ΔpurK, ΔpurL, ΔpurD) in glucose minimal medium, supplemented with either (i) CAA, (ii) the synthetic amino acid mix (SAA) or (iii) the respective essential metabolite (Supplementary Figure 3). As predicted, all mutants, were able to grow only in media supplemented either with CAA or the essential metabolite. This analysis demonstrated that CAA is a complex mixture with several components other than amino acids present in relevant physiological concentrations.

Validation of the FIA-Q-TOF method for amino acid quantification

To extract absolute amino acid concentrations from our FIA-TOF measurements, we used calibration curves generated from external amino acid standards. Specifically, we prepared a mix of all amino acids at predefined concentrations, and measured corresponding MS intensities of several dilutions in M9 glucose minimal medium. Absolute amino acid concentrations were estimated by comparing peak intensities measured in the culture supernatant samples with intensities of the external standards. To validate our method, we compared the amino acid concertation estimates in CAA from FIA-TOF, with a gold standard method for amino acid quantification, i.e. the ACCQ-Tag Ultra Derivatization Kit.

The comparison from the two methods demonstrated excellent correlation (p−value =4.7093e−12) and an average coefficient of variance in line with traditional accepted analysis methods (Supplementary Figure 5). Obvious outliers, were unstable amino acids such as glutamine, and the two isomers leucine and isoleucine, which cannot be distinguished by FIA-TOF analysis. With respect to other methodologies that quantify amino acids, our approach provides a comparable accuracy, but is capable of a higher throughput (~1 vs 35 min per sample quantification), sensitivity and simpler sample preparation.

Supplementary Figures

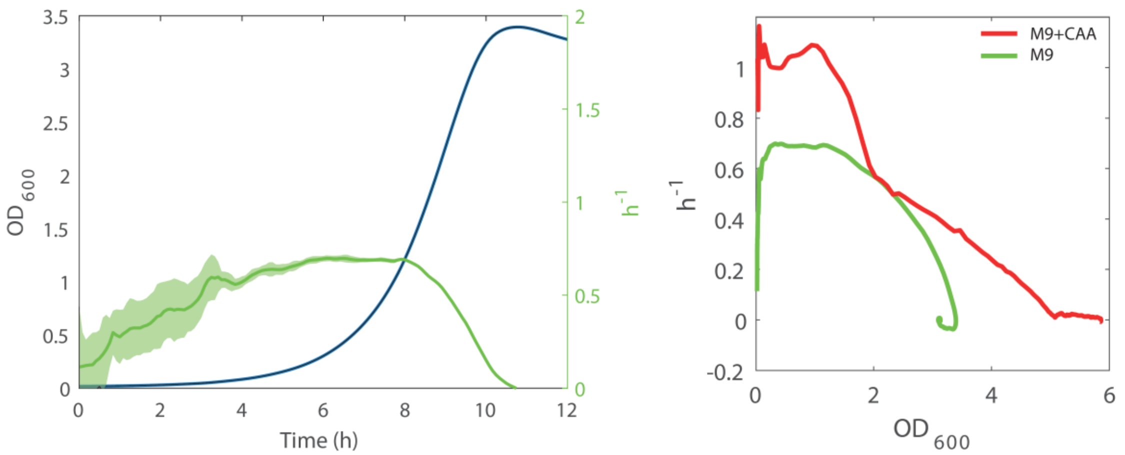

Supplementary Figure 1: Growth curve of E. coli in M9 glucose. Left Panel) OD_{600} (blue line) and instantaneous growth rate (green line) of an *E. coli* culture growing in M9 glucose minimal medium. Shaded region represents standard deviation of growth rate estimates across 4 biological replicates. Right Panel) Instantaneous growth rate of an *E. coli* culture growing in M9 glucose minimal medium (green line) and M9 glucose minimal medium supplemented with casamino acids (red line) against OD_{600}.

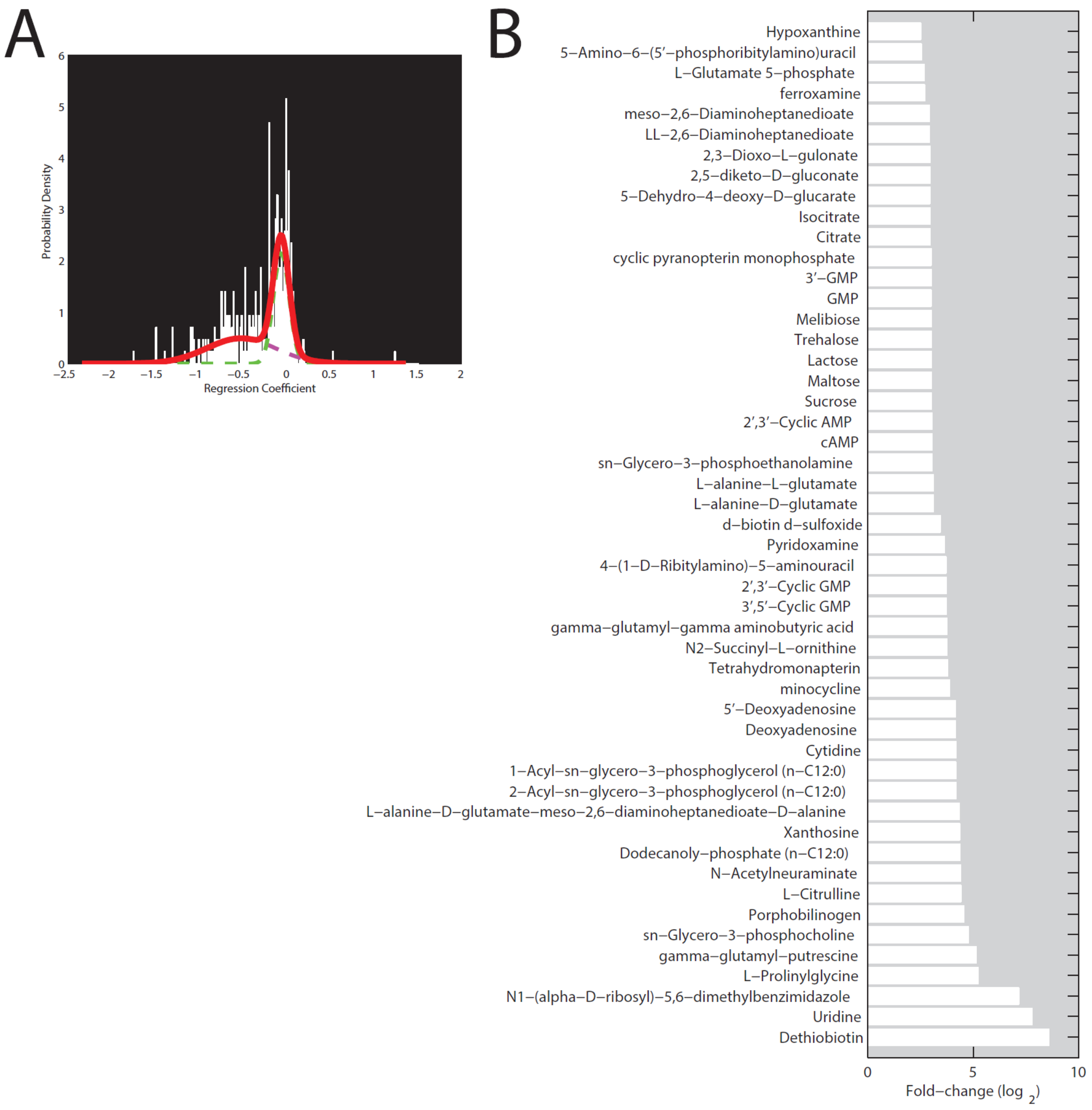

Supplementary Figure 2: Characterization of CAA components. A) To demonstrate and verify the presence of a detected metabolite in the CAA mixture, we prepared a series of 6 CAA dilutions, from 5 to 0.15 g/L, and determined the proportionality between measured intensities and dilution by linear fitting. A) shows the distribution of regression coefficients between CAA dilutions and measured intensities for the 427 detected metabolites. A mixed 2 normal distribution was fitted (red line) and only those compounds with a regression coefficient higher than the mean of the background distribution (green dashed line) plus 2 times the estimated standard deviation were retained and considered. B) Out of the 167 metabolites that exhibited a significant dependency between CAA dilution and measured intensity (pvalue≤0.05), this list displays those 50 metabolites with the highest intensity readouts normalized to a reference sample of M9 minimal glucose medium, which had been supplemented with a pure synthetic amino acid mix (SAA).

	ΔBioA	ΔBioB	ΔBioD	ΔPurC	ΔPurE	ΔPurH	ΔPurK	ΔPurL	ΔPurD	ΔPyrE
**CAA**	✔	✔	✔	✔	✔	✔	✔	✔	✔	✔
**SAA**	✖	✖	✖	✖	✖	✖	✖	✖	✖	✖
**Bio/Xan/Uri**	✔	✔	✔	✔	✔	✔	✔	✔	✔	✔
**M9**	✖	✖	✖	✖	✖	✖	✖	✖	✖	✖

Supplementary Figure 3: Conditional essentiality in M9 and M9 + CAA. We verified the presence of metabolites other than amino acids in the CAA mix, by testing the phenotype of single gene knockouts from the Keio collection [4], auxotrophic for uridine (ΔpyrE), biotin (ΔbioA, ΔbioB, ΔbioD) or xanthine/xanthosine (ΔpurC, ΔpurE, ΔpurH, ΔpurK, ΔpurL, ΔpurD), respectively. This set of mutants were grown in triplicates overnight in a LB rich medium, before cells were washed 3 times and used to inoculate M9 glucose minimal medium, supplemented with (i) SAA, (ii) the respective essential metabolite, or (iii) CAA. All mutants showed the expected phenotypes. Specifically, significant growth (i.e. $OD_{600} \geq 0.2$, above the initial value of 0.05 at inoculation) was detected after 24 and 48 hours only in the M9 medium supplemented either with CAA or with the respective essential metabolite.

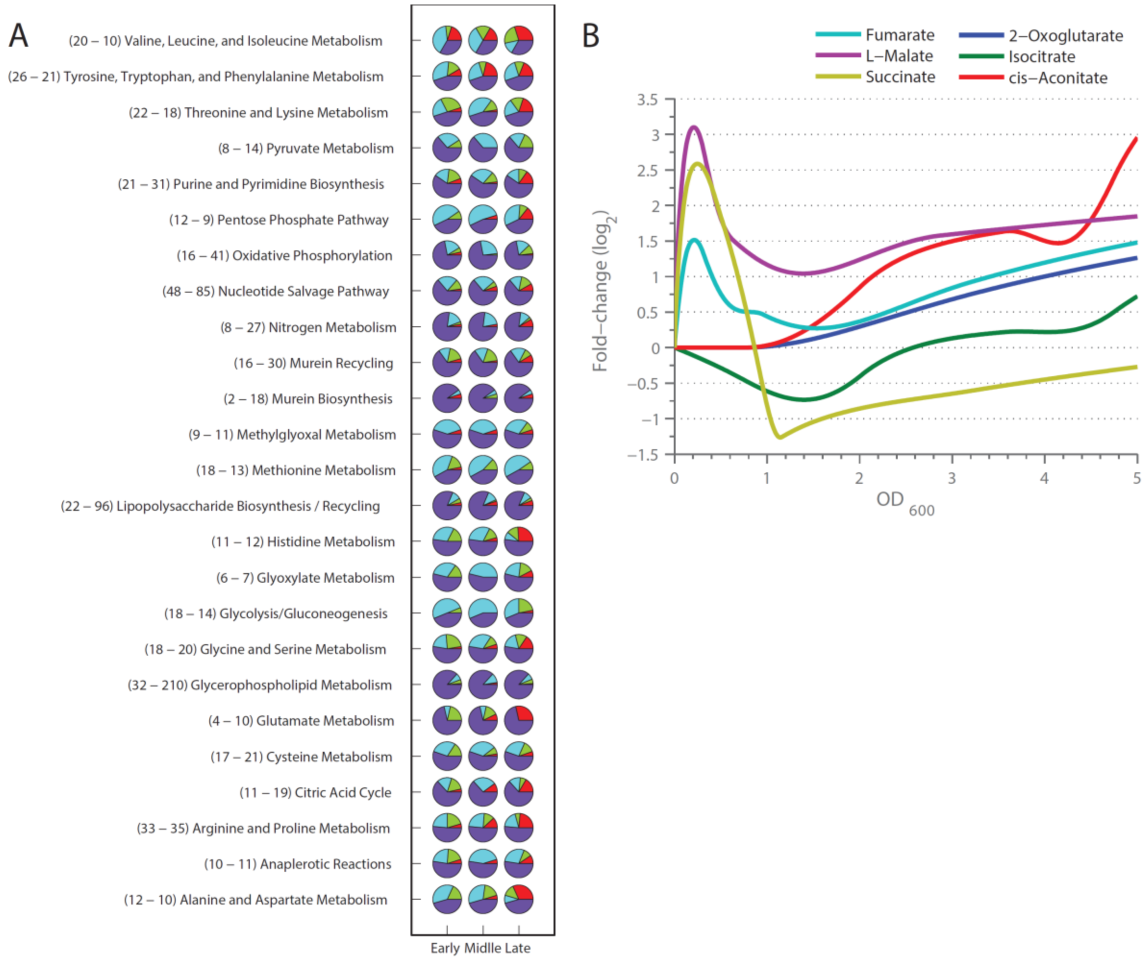

Supplementary Figure 4: Secretion and consumption patterns of metabolites during growth of an *E.coli* culture in M9 glucose minimal medium supplemented with CAA. A) For each metabolic pathway (as defined in the metabolic model [5]), metabolites were grouped in secreted (red), consumed (green), unchanged (light blue) and not-detected (dark blue). To this end, we calculated the relative percentage of metabolites that exhibit changes larger than an arbitrary cutoff of 0.5 in $\log_2$ scale. This grouping was performed for the early (OD_{600} up to 1), middle (OD_{600} between 1 and 2.5) and late (OD_{600} between 2.5 and 4.5) growth phases. This analysis revealed complex exchange patterns for metabolic intermediates of several pathways, and frequent accumulation in the medium during the late growth phase. It is worth noting that while, extensive overflow metabolism has been reported to serve as a general homeostasis mechanisms in *E.coli* [6] [7], its impact on the total cell economy is still poorly understood. Interestingly, intermediates of metabolic pathways with a high protein cost [8], such as methionine biosynthesis and glycolysis, did not exhibit any overflow metabolism. B) Relative changes in the extracellular concentration of TCA cycle intermediates.

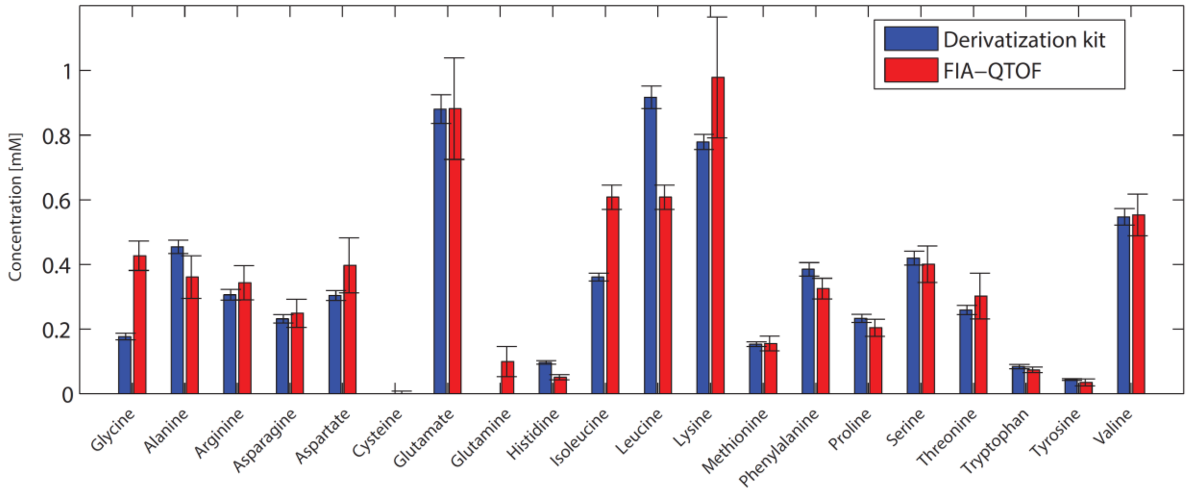

Supplementary Figure 5: Amino acid concentration estimates using our non-targeted mass spectrometry approach and a standard method (AccQ-Tag Ultra Derivatization Kit by Waters). The error bars are the standard deviations of 3 independent measurements.

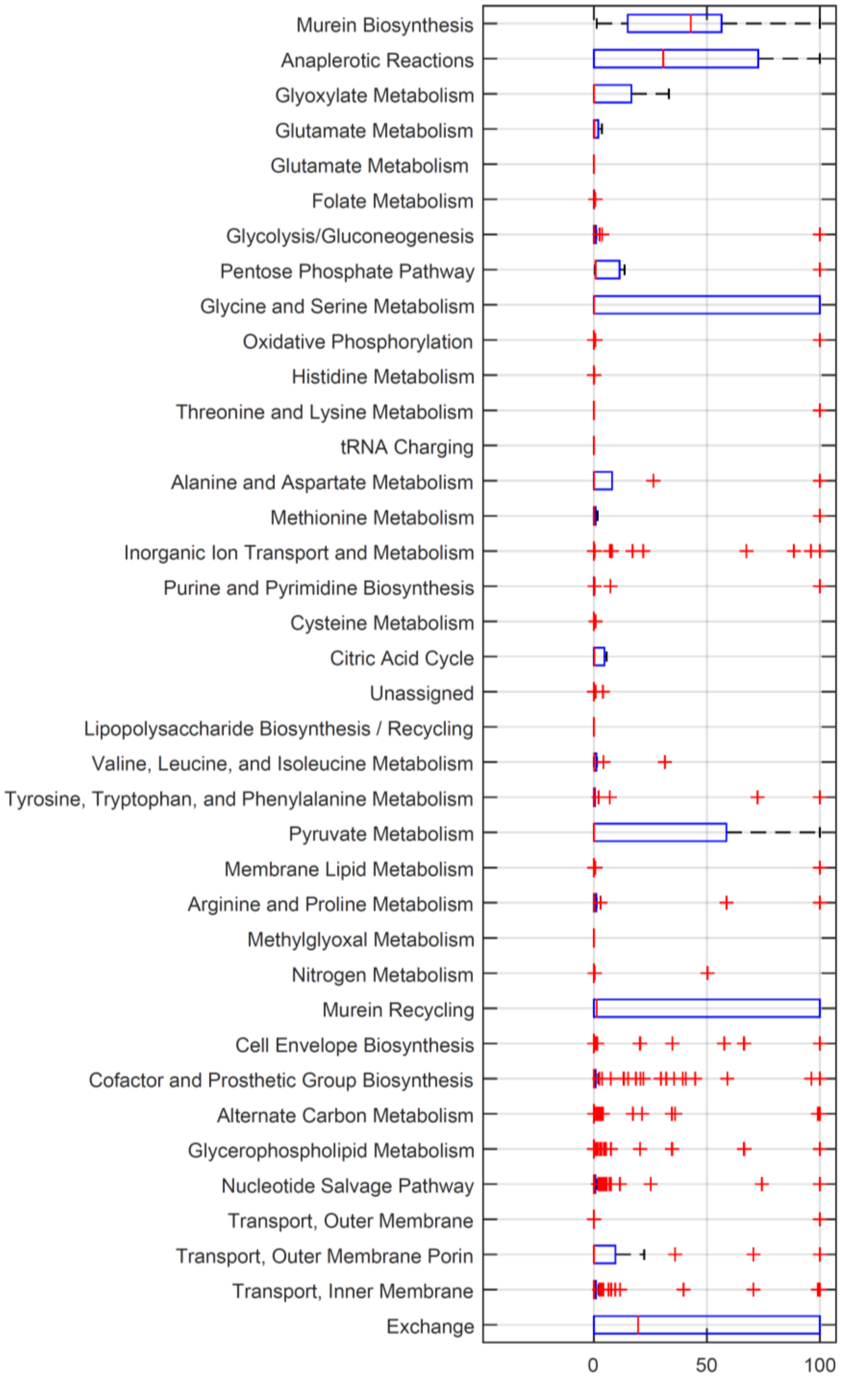

Supplementary Figure 6: FVA analysis. The solution space depends on the model's degrees of freedom and is typically explored using Flux Variability Analysis (FVA), which estimates the minimum and maximum value of each reaction in the proximity of the global optimum. For each reaction in the model we calculated coefficients of variance (CV) from FVA analysis. Specifically, we calculated the absolute values of the ratio between the deviations of minimum/maximum flux values from the optimal flux solution, and optimal flux estimate (i.e. $\max(|(v_{min}-v_{opt})/\ v_{opt}|,|(v_{max}-v_{opt})/\ v_{opt}|)$). The maximum of the two absolute values was retained. Any reaction with min/max flux different from zero and optimal flux equal to 0, were assigned the maximum CV of 100%. For each pathway in the model, boxplots represent the distribution of estimated CVs. Pathways are defined according to annotated subsystems in the genome scale model of *E. coli* [5].

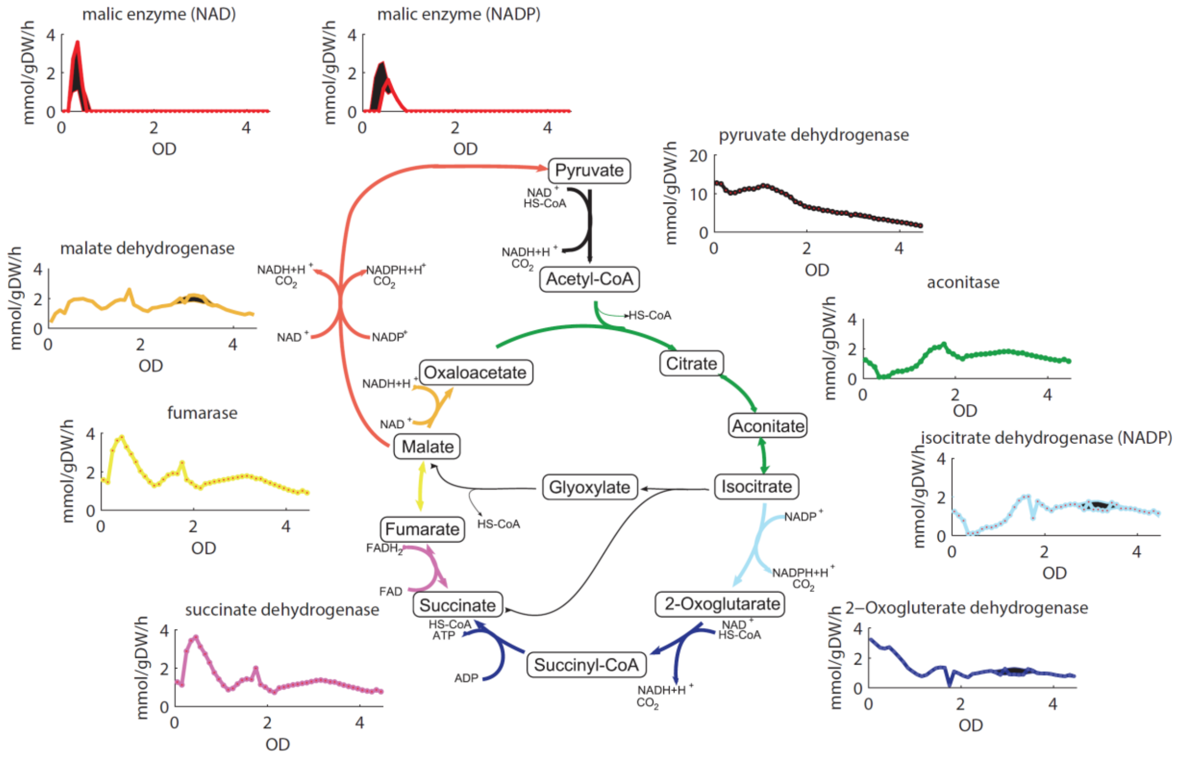

Supplementary Figure 7: Time dependent map of fluxes in TCA cycle. Subplots represent fluxes through TCA cycle reactions, inferred by our dynamic FBA model. FVA results show the feasible space of optimal solutions (black areas). Intermediates in the non-oxidative branch of TCA are initially secreted and then immediately taken up (e.g. succinate).

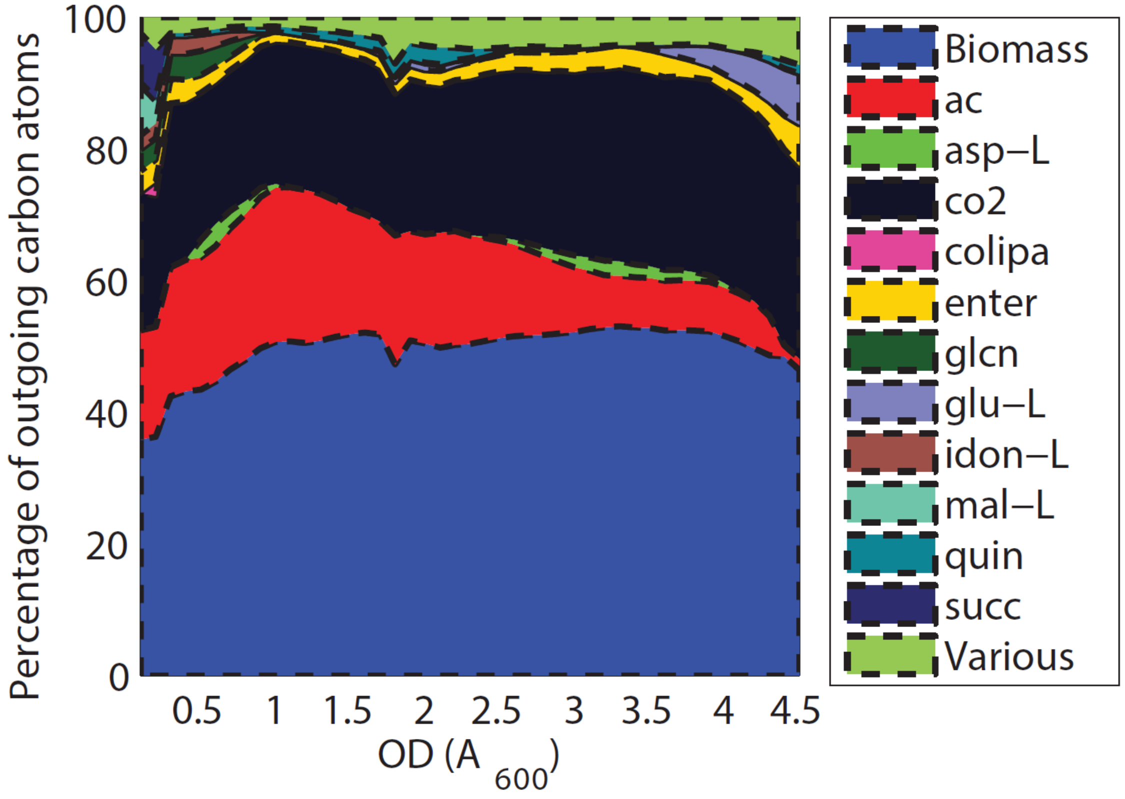

Supplementary Figure 8: Carbon balance. The total outgoing flux of carbon at each given time is calculated by multiplying the number of carbon atoms for each compound and the respective estimated secretion flux. The relative percentage is shown. Only those metabolites contributing for at least 2% of the total outgoing carbon are listed. The remaining compounds are grouped as "Various". Correspondence between metabolite IDs in the legend and metabolite names can be found in the Supplementary Supplementary Data 1.

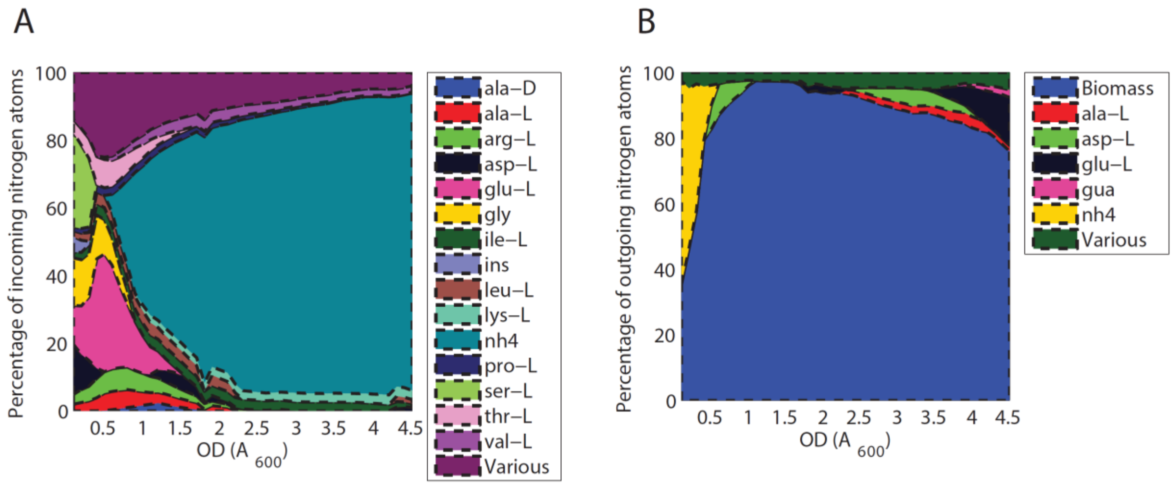

Supplementary Figure 9: Nitrogen balance. The total (A) incoming and (B) outgoing flux of nitrogen at each given time is calculated by multiplying the number of nitrogen atoms for each compound and the respective estimated uptake/secretion flux. The relative percentage is shown. Only those metabolites contributing for at least 2% of the total incoming/outgoing nitrogen are listed. The remaining compounds are grouped as "Various". Correspondence

between metabolite ids in the legend and metabolite names can be found in the supplementary Supplementary Data 1.

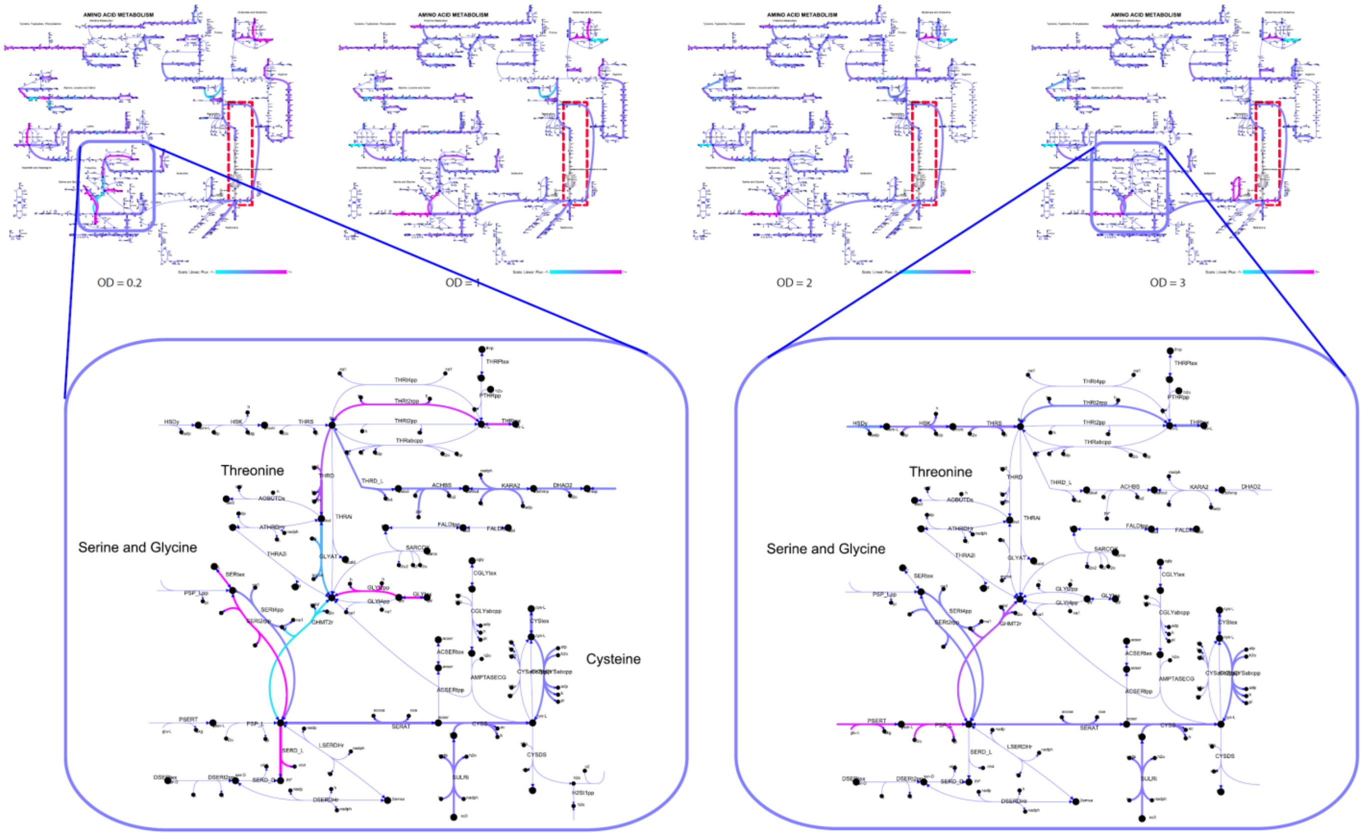

Supplementary Figure 10. Predicted fluxes in the degradation pathways of Serine and Glycine at early and mid-exponential phase. Maps were generated using model-based flux predictions and the Cobra Toolbox display function. Flux values are normalized such that min/max are comprised within -1/1.

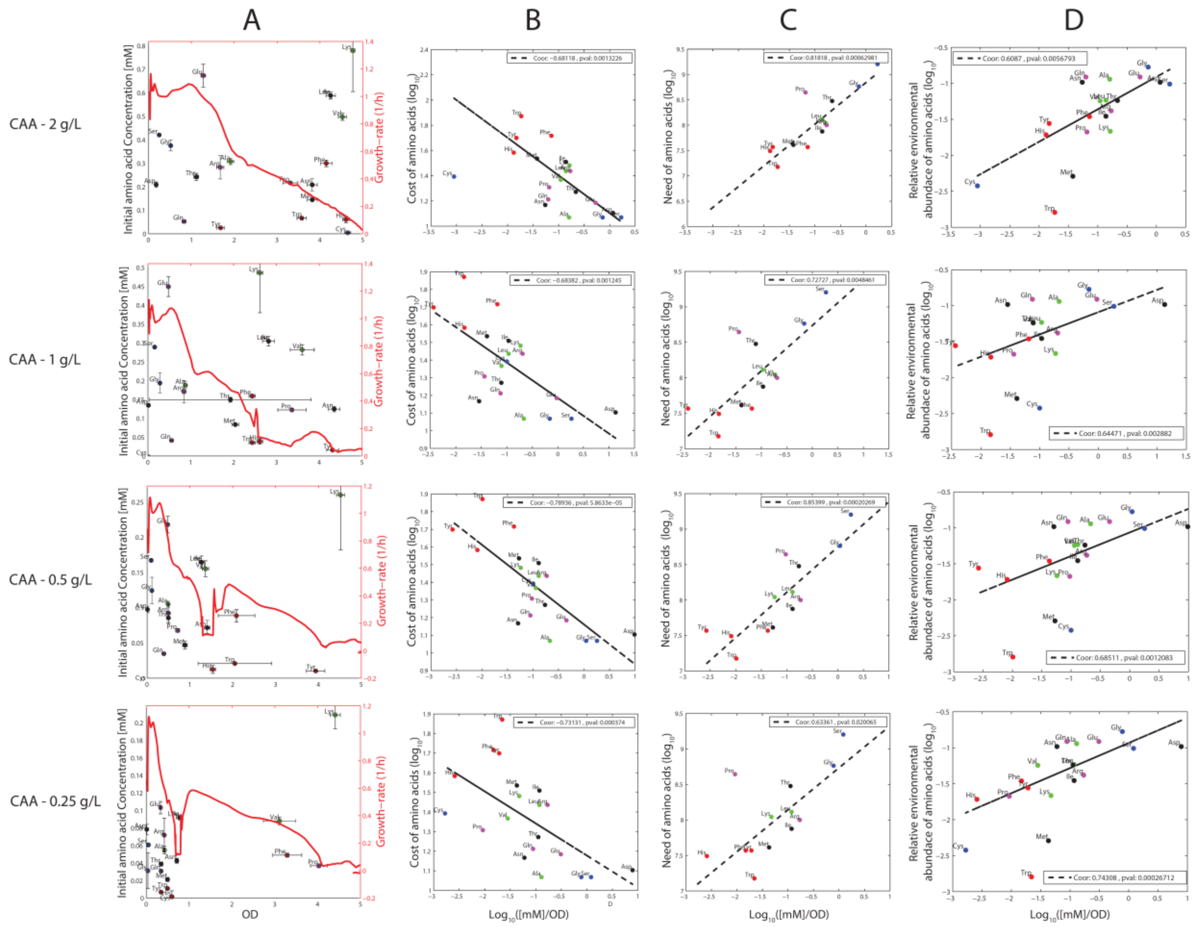

Supplementary Figure 11: Uptake rates of amino acids at different initial CAA concentrations. Here we monitored instantaneous growth rate and amino acid depletion during growth on glucose M9 in the presence of four different CAA concentrations: 2, 1, 0.5 and 0.25 g/L. A) Each dot represents an amino acid, while the red line is the instantaneous cellular growth rate. For each amino acid, the initial concentration is related to the OD_{600} at which the amino acid is depleted. B) For each amino acid, its metabolic cost – number of high-energy phosphate bonds required for biosynthesis [9] - is compared to the average amount of amino acid consumed per unit change in OD_{600}, calculated as the ratio between initial amino acid concentration and culture OD_{600} at time of depletion as shown in (A). C) For 14 amino acids, experimental estimates of the number of extracellular amino acids needed to generate a cell reported in [10] is compared to the average amount of amino acid consumed per unit change in OD_{600}. D) For each amino acid the average relative amino acid concentration across multiple environments reported in [11] is compared to the average amount of amino acid consumed per unit change in OD_{600}.

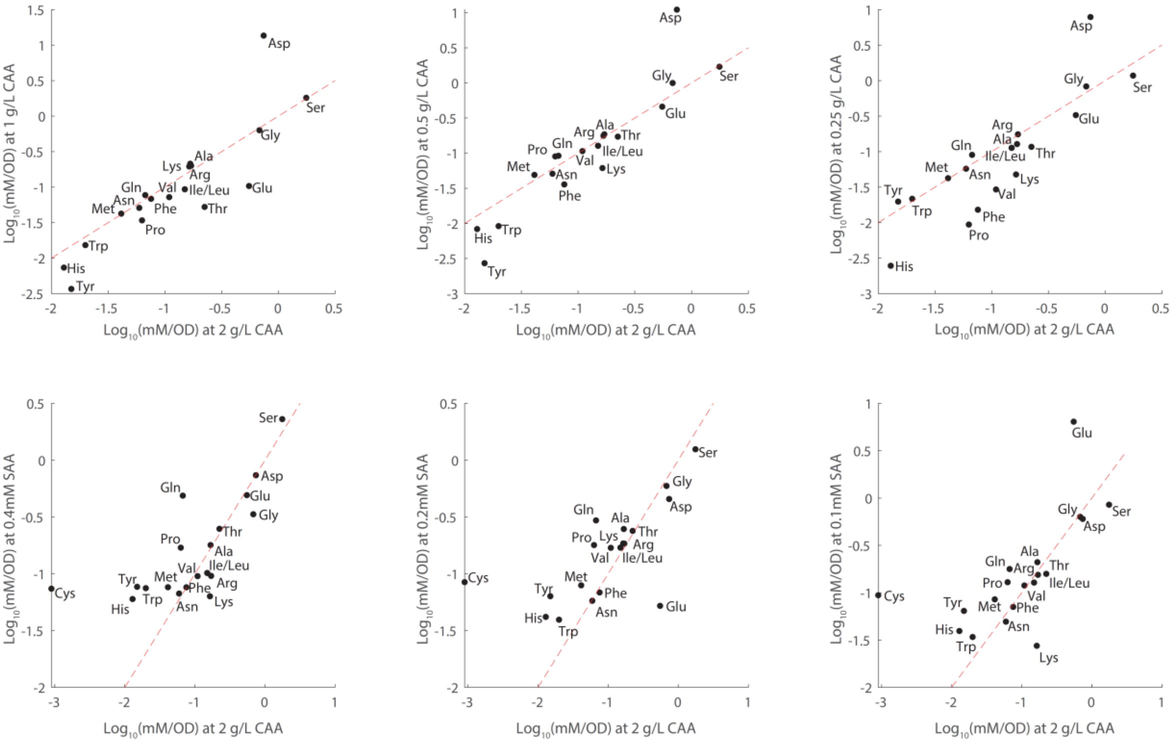

Supplementary Figure 12: Correlation between amino acid metabolism across different initial concentrations. Here the average amount of amino acid consumed per unit change in OD_{600}, determined with an initial concentration of 2g/L CAA is compared to the experimental estimates made with 1, 0.5, 0.25 g/L of CAA and synthetic mixes of amino acids, where all are present at identical concentrations of 0.4, 0.2 and 0.1 mM, respectively.

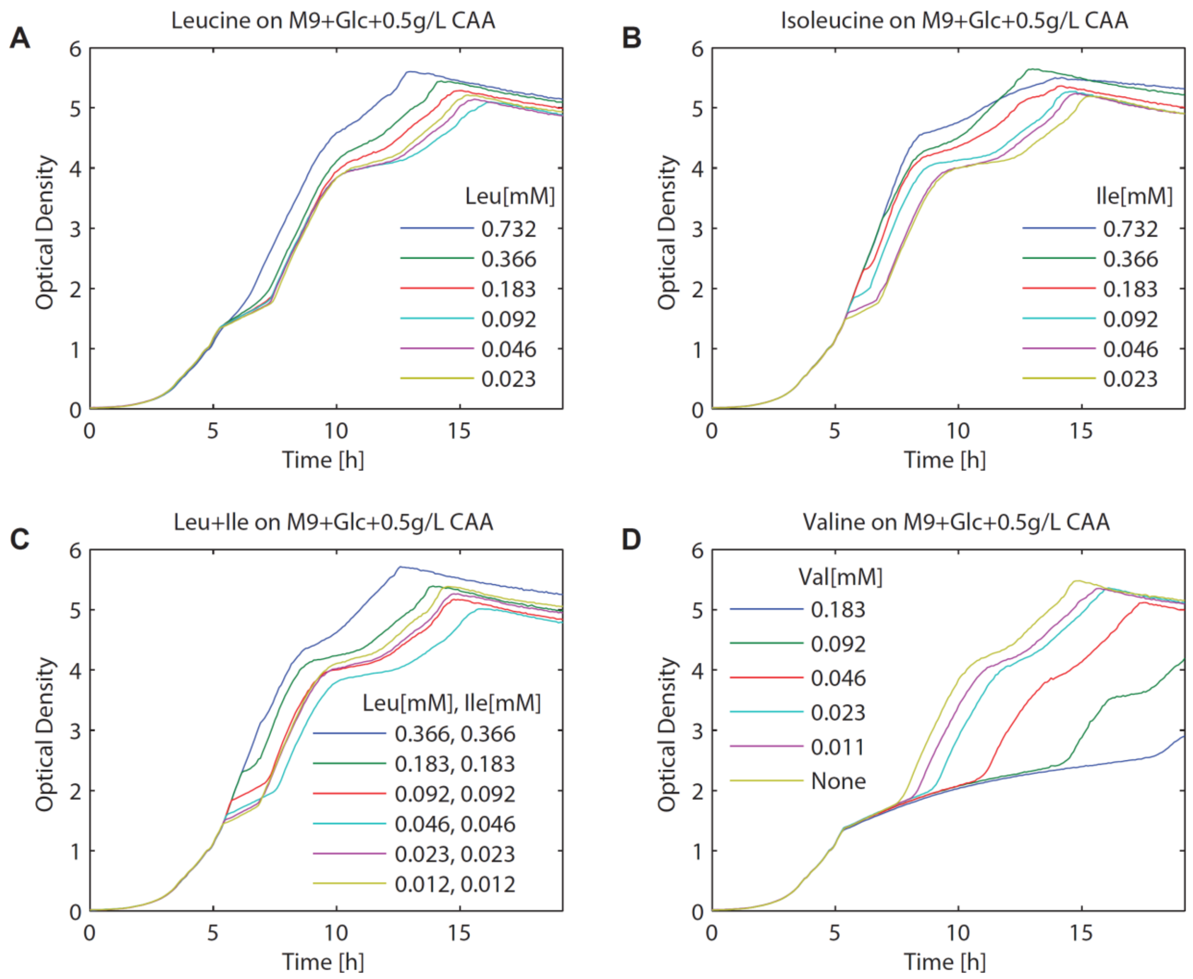

Supplementary Figure 13: Functional dependency between valine and iso-/leucine. A) Growth curves of *E. coli* growing in M9 glucose + 0.5 g/L of CAA supplemented with different concentrations of leucine, B) isoleucine, C) leucine + isoleucine, D) valine.

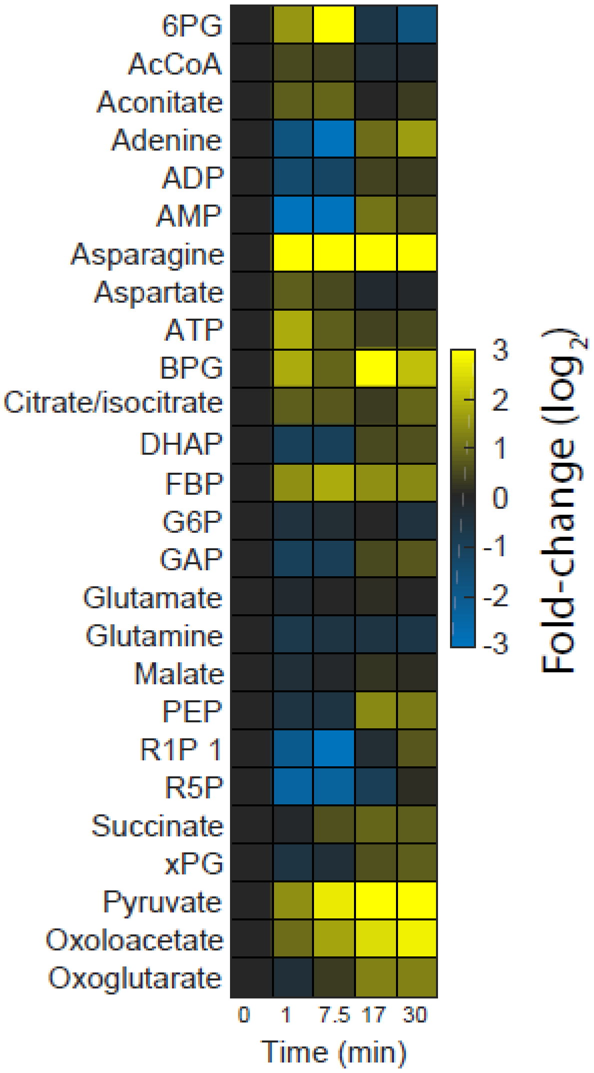

Supplementary Figure 14. LC-MS measurements of intracellular metabolite changes upon CAA supplementation. Targeted LC-MS mass spectrometry was used to quantify relative changes in the abundance of 26 metabolites in *E. coli* upon supplementation of glucose M9 medium with 2g/L of CAA. Relative changes 1, 7.5, 17 and 30 minutes after spiking the medium with CAA were measured and reported as fold changes relative to metabolite levels immediately before addition of CAA. This data validates non-targeted profiles, in particular the rapid and strong accumulation of pyruvate and oxaloacetate. In addition, we found strong changes (absolute fold-change>2) of intermediates in pentose phosphate pathway, such as 6-phospho gluconate (6PG), ribose-5 phosphate (R5P) and ribose-1 phosphate (R1P1), taking place approximately within the first 10 minutes after CAA supplementation.

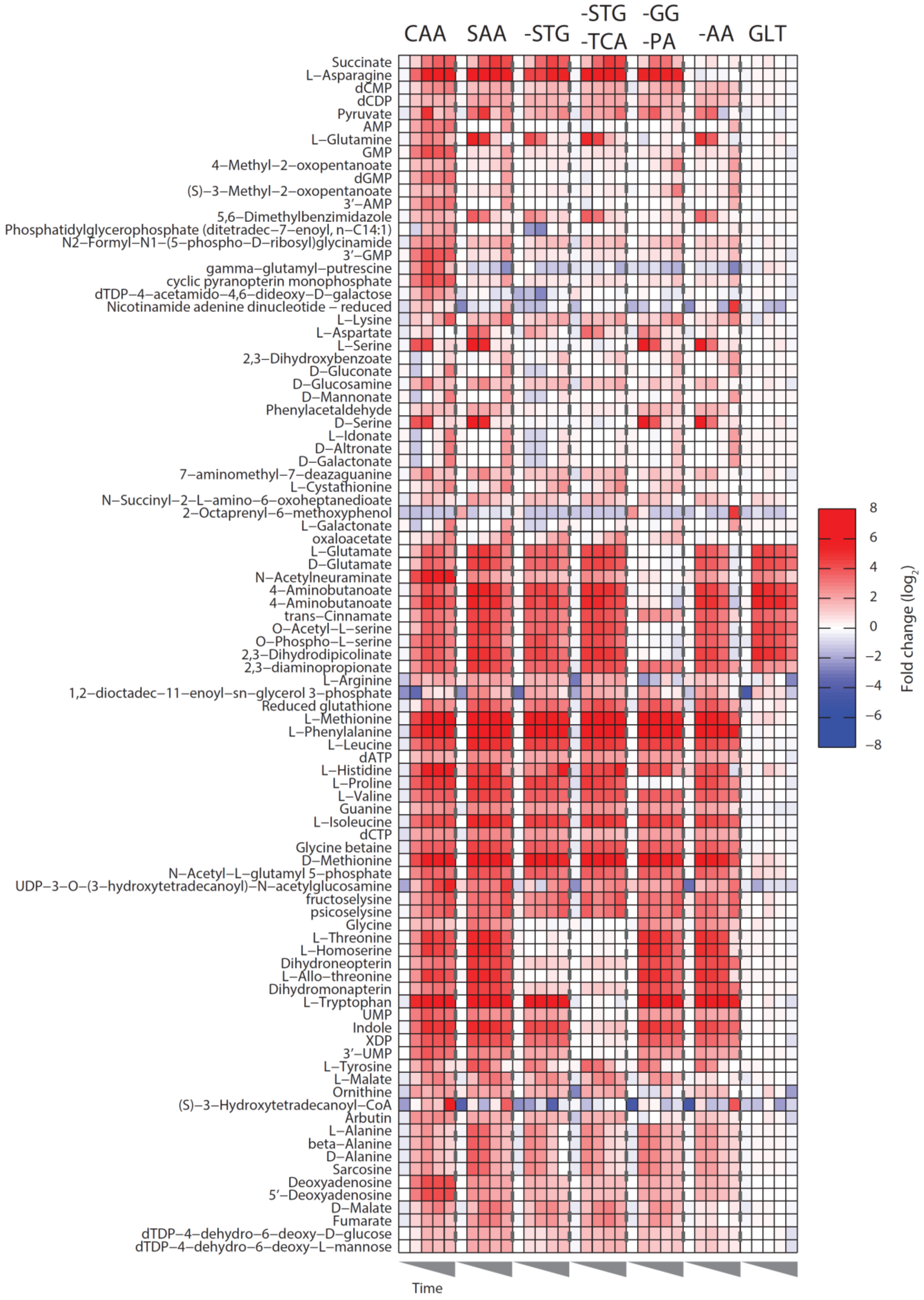

Supplementary Figure 15: Metabolome changes induced upon addition of different amino acids mixtures. Heatmap of metabolite fold changes (log2) at 0, 15, 30, 60 and 90 min after addition of different mixtures of amino acids: casamino acids (CAA), synthetic amino acid mix (SAA), SAA deprived of amino acids that can be degraded into pyruvate (i) threonine, glycine

and serine (SAA – STG) or (ii) threonine, glycine, serine, tryptophan, cysteine and alanine (SAA - STGTCA), or α-ketoglutarate (iii) glutamate, glutamine, proline and arginine (SAA – GGPA), or oxaloacetate (iv) aspartate and asparagine (SAA - AA), and 0.125 g/L of glutamate (GLT). Only metabolites that exhibit an absolute fold change greater than 4 in at least one condition are shown (Supplementary Data 2).

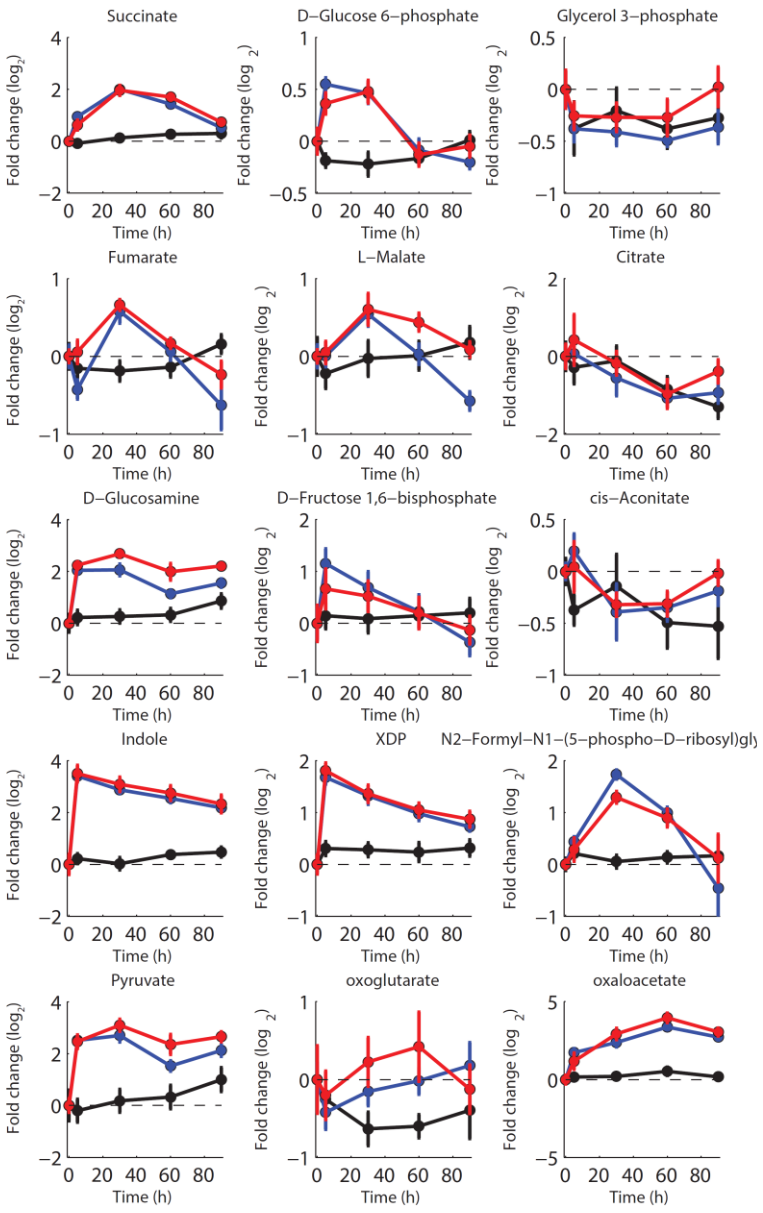

Supplementary Figure 16: Validation of whole cell broth profiles. Relative changes in intracellular metabolite levels using a fast filtration protocol to remove the supernatant from *E. coli* cells perturbed with CAA (red) and SAA (blue) and the control (black). Here we report the dynamic profiles of few key selected metabolites, the full dataset can be found in Supplementary Data 2.

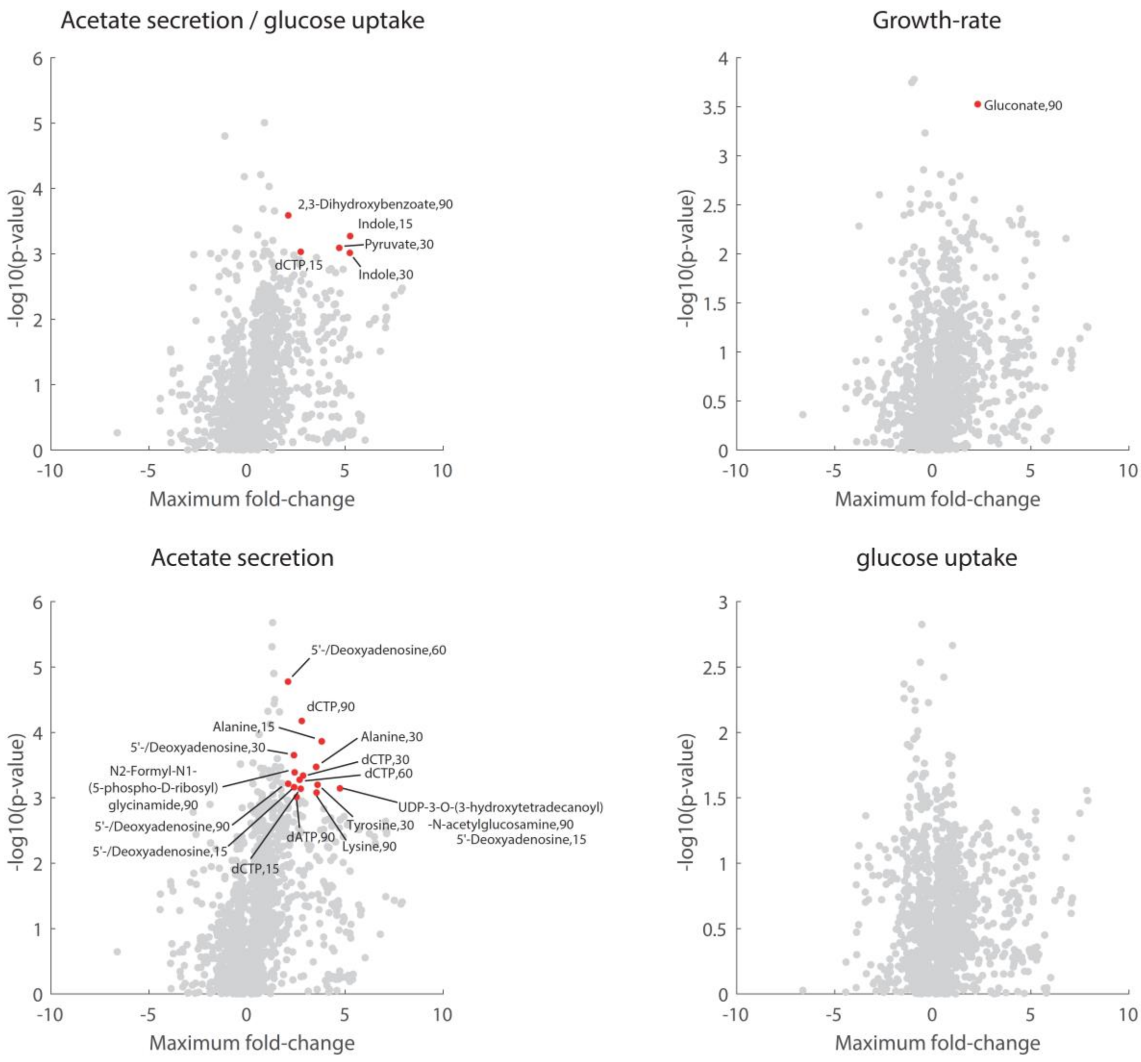

Supplementary Figure 17. Correlation anlysis between metabolite levels and glcuose uptake, acetate secretion and growth rates. For each metabolite, we calculate the Pearson correlation between relative changes at 15, 30, 60 and 90 minutes after perturbation with the 8 different amino acids mixes, and the ratio between acetate and glucose, glucose uptake, acetate secretion and growth rates. Each dot corresponds to one metabolite and one time point. Maximum fold change across the eight conditions at a specific time point is plotted against the correlation p-value. Only metabolites with a maximum fold change greater than 2 in log2 scale and p-value≤0.001 are selected and highlighted in red. Lables indicate metabolite name and time (min) after addition of amino acids.

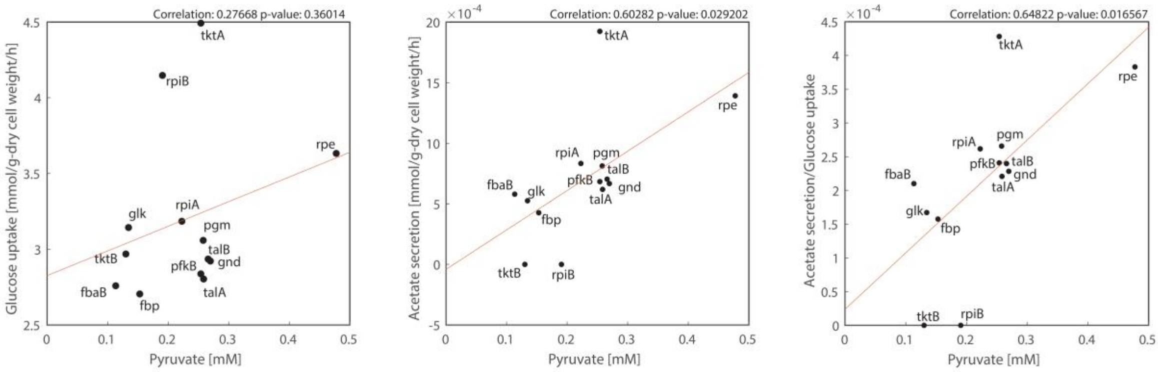

Supplementary Figure 18. Correlation between pyruvate levels and acetate secretion vs glucose uptake in genetically modified *E. coli* strains. Here we use previolsy published data [12] to compare intracellular pyruvate levels with (left panel) glucose uptake, (central panel) acetate secretion and (right panel) acetate seceretion vs glucose uptake in 13 *E. coli* knckout strains. Consistent with our results we found the strongest correaltion between pyruvate levels and the ratio between acetate sectretion and glucose consumption.

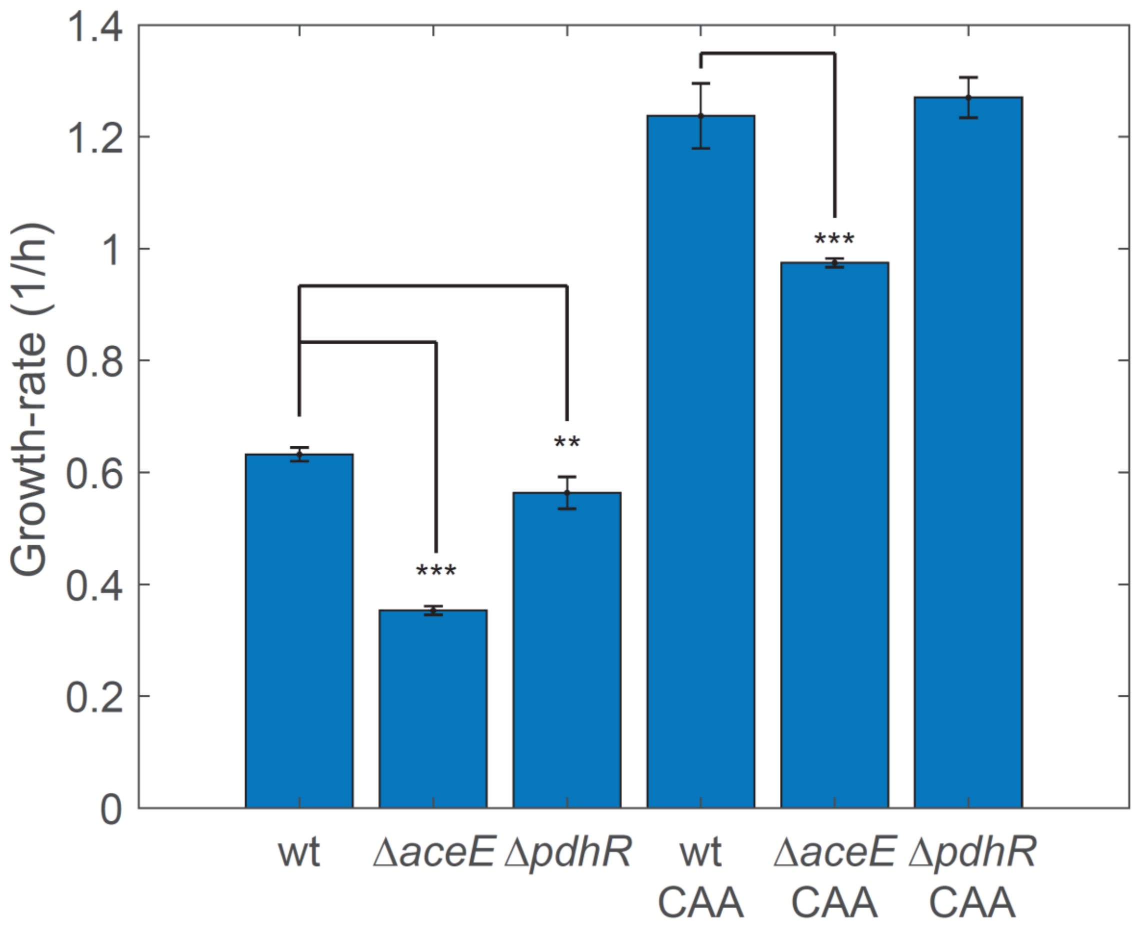

Supplementary Figure 19. Functionality of PdhR in M9 with CAA. Growth rates of wild type *E.coli*, *ΔaceE* and *ΔpdhR* in glucose M9 and glucose M9 + CAA. As expected *ΔaceE* grows significantly slower in both media (*** p-value<0.001), while *ΔpdhR* exhibits a mild but significant (** p-value<0.01) reduction in growth rate only in the absence of CAA.

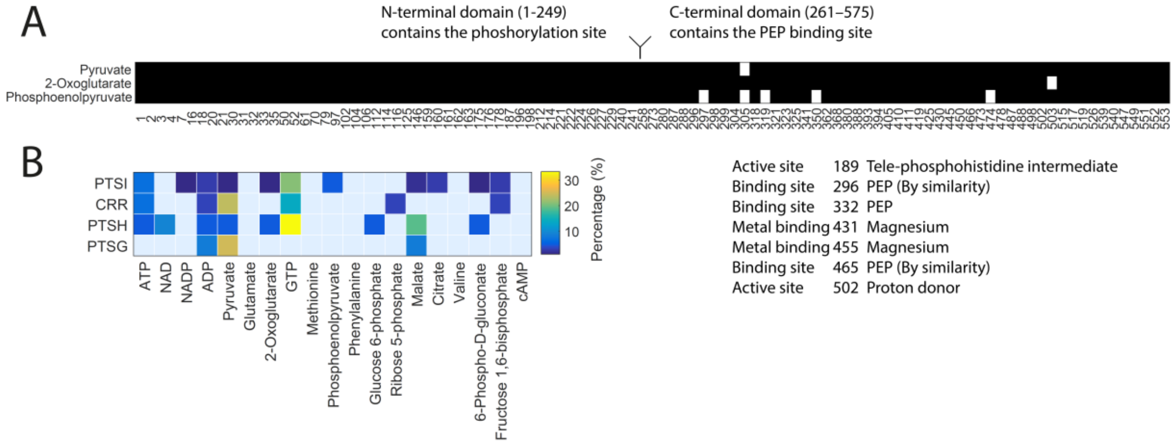

Supplementary Figure 20. Limited Proteolysis Analysis of PTS complex . A) We reanalyzed previously published limited proteolysis data[13], systematically mapping conformational changes in *E. coli* proteome upon incubation with 20 different metabolites, including pyruvate. In the heatmap we report all peptides mapping to the PtsI protein, sorted by their initial position. Only peptides that were found to undergo significant (q-value<=0.01) conformational changes upon incubation with pyruvate, oxoglutarate and phosphoenolpyruvate (PEP) are highlighted (e.g. white). Annotation of known PtsI binding sites is reported on the lower right corner. B) The heatmap summarize the conformational changes induced by the 20 metabolites tested in [13] for the proteins of the PTS complex: PtsI, Crr PtsH and PtsG. For each metabolite and protein, we report the percentage of detected peptides that exhibit significant (q-value<=0.01) conformational changes. We found that not only PtsI undergoes significant (q-value<=0.01) conformational changes (panel A), but that all protein subunits of the PTS system (PtsG, Crr, PtsH and PtsI) exhibit conformational changes on multiple sites.

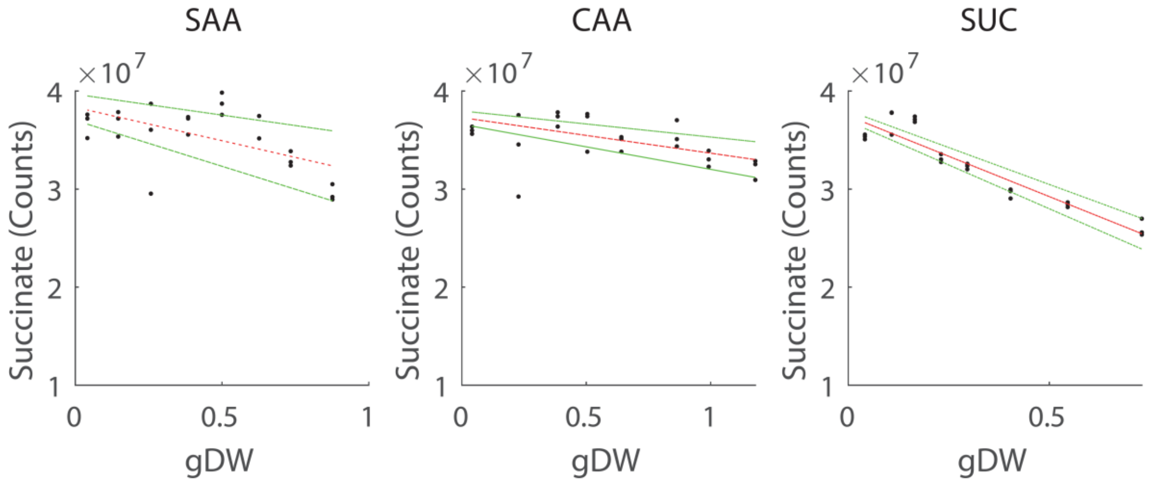

Supplementary Figure 21. Succinate uptake rates. Measured succinate intensitites against gram of dry biomass (gDW) during growth in succinate minimla medium (SUC), supplemented with 2g/L CAA or a synthetic mix of amino acids (SAA) consisting of the same amino acids and concentrations measured in 2g/L CAA. Red dashed line are the best linear fits and green dashed line illustrate the 95% confidence interval from fitting estimates.

Supplementary References

1. Marmorstein, R. Q., Joachimiak, A., Sprinzl, M. & Sigler, P. B. The structural basis for the interaction between L-tryptophan and the Escherichia coli trp aporepressor. *J. Biol. Chem.* **262**, 4922–4927 (1987).
2. Foster, J. W. Escherichia coli acid resistance: tales of an amateur acidophile. *Nat. Rev. Microbiol.* **2**, 898–907 (2004).
3. Fuhrer, T., Heer, D., Begemann, B. & Zamboni, N. High-throughput, accurate mass metabolome profiling of cellular extracts by flow injection-time-of-flight mass spectrometry. *Anal. Chem.* **83**, 7074–7080 (2011).
4. Baba, T. *et al.* Construction of Escherichia coli K-12 in-frame, single-gene knockout mutants: the Keio collection. *Mol. Syst. Biol.* **2**, 2006.0008 (2006).
5. Orth, J. D. *et al.* A comprehensive genome-scale reconstruction of Escherichia coli metabolism--2011. *Mol. Syst. Biol.* **7**, 535 (2011).
6. Reaves, M. L., Young, B. D., Hosios, A. M., Xu, Y.-F. & Rabinowitz, J. D. Pyrimidine homeostasis is accomplished by directed overflow metabolism. *Nature* **500**, 237–241 (2013).
7. Paczia, N. *et al.* Extensive exometabolome analysis reveals extended overflow metabolism in various microorganisms. *Microb. Cell Factories* **11**, 122 (2012).
8. Li, G.-W., Burkhardt, D., Gross, C. & Weissman, J. S. Quantifying Absolute Protein Synthesis Rates Reveals Principles Underlying Allocation of Cellular Resources. *Cell* **157**, 624–635 (2014).
9. Akashi, H. & Gojobori, T. Metabolic efficiency and amino acid composition in the proteomes of Escherichia coli and Bacillus subtilis. *Proc. Natl. Acad. Sci.* **99**, 3695–3700 (2002).
10. Mee, M. T., Collins, J. J., Church, G. M. & Wang, H. H. Syntrophic exchange in synthetic microbial communities. *Proc. Natl. Acad. Sci.* **111**, E2149–E2156 (2014).
11. Moura, A., Savageau, M. A. & Alves, R. Relative Amino Acid Composition Signatures of Organisms and Environments. *PLoS ONE* **8**, e77319 (2013).
12. Ishii, N. *et al.* Multiple high-throughput analyses monitor the response of E. coli to perturbations. *Science* **316**, 593–597 (2007).
13. Piazza, I. *et al.* A Map of Protein-Metabolite Interactions Reveals Principles of Chemical Communication. *Cell* **172**, 358-372.e23 (2018).
