# Supplementary material for: Regulatory mechanisms underlying coordination of amino acid and glucose catabolism in Escherichia coli
Source: Nat Commun. 2019 Jul 26;10:3354. doi: 10.1038/s41467-019-11331-5 (PMC6659692; doi:10.1038/s41467-019-11331-5)
Supplement: Supplementary file 3 — Description of Additional Supplementary Files [file 41467_2019_11331_MOESM3_ESM.pdf]

Description of Additional Supplementary Files

File Name: Supplementary Data 1
Description:

- **Metabolite Abbreviations:** List of metabolite abbreviations as reported in the genome-scale metabolic model, and here used in Figure 3 of the main text.
- **Estimated fluxes:** Estimates of time-dependent fluxes (mmol/gDW/h) estimated from our FBA-like analysis.
- **Fluxes max:** For each reaction in the model we performed flux variability analysis to estimate the maximum value fulfilling global flux optimality.
- **Fluxes min:** For each reaction in the model we performed flux variability analysis to estimate the minimum value fulfilling global flux optimality.
- **Absolute Meas fluxes:** Absolute measurements of glucose, acetate and amino acids exchange rates.
- **Absolute Meas fluxes LB:** Lower bound of absolute measurements of glucose, acetate and amino acids exchange rates.
- **Absolute Meas fluxes UB:** Upper bound of absolute measurements of glucose, acetate and amino acids exchange rates.
- **Relative Meas fluxes:** Estimated fluxes from relative non-targeted MS data.
- **Raw data:** Non-targeted metabolomics profiles before using multivariate adaptive regression splines.
- **Fitted data:** Results of the multivariate adaptive regression splines.

File Name: Supplementary Data 2
Description:

- **AA perturbations:** (Whole culture broth extraction protocol) non-targeted MS profiles of metabolites in response to perturbations of cells with different amino acids mixes. Data are averaged over 3 biological replicates.
- **AA perturbations std:** Standard deviation of non-targeted MS profiles of metabolites in response to perturbations of cells with different amino acids mixes.
- **LC-MS CAA intracellular:** Targeted MS profiles of intracellular metabolites in response to supplementation of cell with CAA for 30 minutes.
- **Intracellular FC CAA & SAA**: (Fast-filtration protocol) non-targeted MS profiles of intracellular metabolites in response to perturbations of cells with CAA or SAA. Data are averaged over 3 biological replicates.
- **Intracellular FC std CAA & SAA:** (Fast-filtration protocol) standard deviation of non-targeted MS profiles of intracellular metabolites in response to perturbations of cells with CAA or SAA.
- **Correlation analysis:** Results from correlation analysis described in figure 5 of the main text and S15 of the supplements.
- **AA uptake rates:** Experimentally determined average uptake rates of amino acids in the 7 mixes.
